# Stroke among Māori in Aotearoa New Zealand and solutions to address persistent inequities

**DOI:** 10.3389/fstro.2023.1248351

**Published:** 2023-08-24

**Authors:** Anna Ranta, Bernadette Jones, Matire Harwood

**Affiliations:** ^1^Department of Medicine, University of Otago, Wellington, New Zealand; ^2^Department of Neurology, Te Whatu Ora Health NZ, Capital, Coast, and Hutt Valley, Wellington, New Zealand; ^3^Department of Primary Care and Rural Health, University of Auckland, Auckland, New Zealand

**Keywords:** stroke, indigenous, Māori, disparities, health equity

## Abstract

M$\bar{\text{a}}$ori, the Indigenous people of Aotearoa New Zealand (Aotearoa), experience stroke at a younger age and at a greater rate than New Zealanders of European ethnicity (NZ Europeans). These disparities have persisted for decades and recent evidence suggests that the gap is widening. M$\bar{\text{a}}$ori also experience reduced access to some key stroke-management interventions and consequently worse post-stroke outcomes compared to non-M$\bar{\text{a}}$ori counterparts. Reasons for the ethnic differences in stroke rates and outcomes include differential exposure to stroke risk factors, differential access to early diagnosis and treatment, and unequal treatment. Recent Aotearoa-based research has suggested that the root causes for these ethnic inequities, including unconscious bias and institutional racism, are likely attributable to Aotearoa's colonial past and related inter-generational sequalae. With recent reforms to the national health system there is now a new mandate to actively move toward a more bicultural approach which emphasizes Indigenous rights, values, priorities, and approaches in healthcare. This presents important opportunities to address the well-described inequities using a genuine partnership model. This paper will discuss the latest evidence around stroke related health disparities affecting M$\bar{\text{a}}$ori, describe existing approaches to address inequitable health outcomes, and present additional novel avenues that are currently being explored.

## Historical context

Aotearoa New Zealand (Aotearoa) is a South Pacific nation. Indigenous M$\bar{\text{a}}$ori had settled in Aotearoa around 1300 AD and Europeans arrived and actively colonized the country in the 18^th^ century (Walter et al., 2017). Aotearoa achieved independence from the British Crown in 1835 but this was superseded in 1840 by Te Tiriti o Waitangi (Te Tiriti) and its English translated version “The Treaty of Waitangi.” Considered Aotearoa's founding document, each version of Te Tiriti had three articles pertaining to protection, governorship and sovereignty although translations do not exactly align (King, 2012; Came et al., 2020). Despite signing Te Tiriti in good faith, M$\bar{\text{a}}$ori rapidly lost ownership of the majority of their land holdings via at best dubious and at worst outright illegal approaches (Came et al., 2020). In addition, active disruption of M$\bar{\text{a}}$ori family units and tribal structures was undertaken by the Crown involving separation of M$\bar{\text{a}}$ori children from their parents and forcibly assimilating them into colonial culture which has resulted in ongoing transgenerational trauma, socioeconomic disadvantage, and health disparities (King, 2012). The historical impacts of colonization can still be seen negatively impacting on health outcomes for M$\bar{\text{a}}$ori, particularly in long-term conditions such as the prevention and management of stroke.

## Stroke incidence in Māori

The best evidence around stroke incidence in M$\bar{\text{a}}$ori comes from the multi-decade Auckland Regional Community Stroke (ARCOS) studies which have recorded stroke incidence every decade since 1980 (Feigin et al., 2015). Throughout these studies M$\bar{\text{a}}$ori stroke incidence has been higher than those with self-determined NZ European ethnicity. In addition, M$\bar{\text{a}}$ori have consistently been shown to experience stroke 10–15 years earlier (59.6 vs. 75.3; *p* < 0.0001 in 2011–2012). Recent findings indicate that the gap is widening with evidence of significant reductions in stroke incidence among NZ Europeans that is not mirrored in M$\bar{\text{a}}$ori (Feigin et al., 2015).

The reason for higher incidence and younger age has been largely attributed to a higher prevalence of stroke risk factors among M$\bar{\text{a}}$ori. For example, smoking, diabetes, and rheumatic fever are more common in M$\bar{\text{a}}$ori compared with New Zealand (NZ) Europeans; and atrial fibrillation is more common among M$\bar{\text{a}}$ori compared with Pacific and Asian ethnicities although similar to NZ Europeans albeit at a younger age (Feigin et al., 2015; Thompson S. G. et al., 2022). The reason for higher prevalence of stroke risk factors has been more difficult to explain. However, the main driver appears to be behavioral and life style related and there is a recent shift from attributing this to individuals' choices (“victim blaming”) to a more holistic view that attributes differential exposure to opportunities and factors that affect health including; education, income, housing and justice (Robson and Harris, 2007).

## Stroke service access in Māori

The recent REGIONS Care study is the most comprehensive stroke study across all of Aotearoa, exploring differences in stroke service access and outcomes by ethnicity and geography adjusting for age, stroke severity, baseline function, and other relevant risk factors (Thompson S. G. et al., 2022). This study found evidence of differential access to best practice stroke services among M$\bar{\text{a}}$ori. For example, M$\bar{\text{a}}$ori were less likely to be cared for in an acute stroke unit (NZ European = 1,423/1,823 (78.1%) vs. M$\bar{\text{a}}$ori 192/273 (70.3%); aOR 0.66 (0.51–0.91) and had trended toward a lower rate of receiving a swallow assessment within 6 h [NZ European 916/1,165 (83.7%) vs. M$\bar{\text{a}}$ori 123/151 (81.5%; aOR 0.69 (0.47–1.00)].

In contrast to other international research involving Indigenous peoples, REGIONS Care did not identify any gaps in accessing stroke reperfusion therapy for M$\bar{\text{a}}$ori: Thrombolysis rate was 12.3% for NZ Europeans and 16.3% for M$\bar{\text{a}}$ori (16.3%); [aOR 0.88 (0.58–1.35)] and thrombectomy rate was 3.4% for NZ Europeans and 4.3% for M$\bar{\text{a}}$ori [aOR 0.67 (0.31–1.47)]. Similarly different from international literature is that presentation within 4 hours of stroke onset was similar among M$\bar{\text{a}}$ori and NZ Europeans: 44.9 vs. 43.8% [aOR 1.01 (0.78–1.32)]. Similar findings were reported by Samuels et al. (2020) all in an Auckland and wider Northern Region study. This study found that M$\bar{\text{a}}$ori, while being younger, experienced a higher thrombectomy intervention rate than other ethnicities and found no difference in process of care metrics, or outcomes (Samuels et al., 2020). It is of interest to note that M$\bar{\text{a}}$ori presented with more severe strokes, as was the case in REGIONS care overall, suggesting the possibility of under-representation of M$\bar{\text{a}}$ori with milder strokes, which could skew data to a higher intervention rate while overlooking potential unmet community need. In contrast to Samuels et al. (2020) a recent Aoteraora national report on reperfusion interventions found evidence for slower door to needle time among M$\bar{\text{a}}$ori not demonstrated by Samuels and colleagues. Thus, the association between adverse reperfusion metrics and M$\bar{\text{a}}$ori ethnicity remains uncertain (Fushida-Hardy et al., 2022).

Some aspects of stroke care considered as part of the REGIONS care project were accessed more readily by M$\bar{\text{a}}$ori compared with NZ Europeans including counseling on smoking cessation, exercise, diet, and what to do if symptoms recur although only the last item reached significance [aOR 1.88 (1.31–2.71)]. There was also a trend for a greater rate of general practitioner review following stroke for M$\bar{\text{a}}$ori compared with NZ Europeans [aOR 1.27 (0.94–1.73)] although no difference in specialist follow-up [0.99 (0.67–1.22)] (Thompson S. G. et al., 2022).

## Stroke outcomes in Māori

Stroke outcomes among M$\bar{\text{a}}$ori have been explored in several studies with varying results. The ARCOS IV incidence study found no significant difference in 1-month case-fatality for M$\bar{\text{a}}$ori in 2011–12 (16.7%) compared with NZ European (19.5%) in the greater Auckland region (Feigin et al., 2015). This study also reported a 1-month M$\bar{\text{a}}$ori case fatality decline between 2002 and 2003 (23.5%) and 2011–12 (16.7%) with a similar pattern observed in NZ Europeans and no significant intergroup difference in rate of decline. Similar patterns were observed for 1-year mortality. This contrasts with the 1-month case fatality reported by Sandiford et al. (2016) which found that M$\bar{\text{a}}$ori 1-month case fatality was greater (16.2%) than NZ Europeans (10.7%) in 2010–2014 and while both cohorts showed a reduction in mortality over the previous 10 years the difference was only significant in the NZ European group (13.4% to 10.7%; vs. 18.2% to 16.2%). Furthermore, after adjusting for demographic variables, service delivery factors, and comorbidities, they reported the outcome disparity had in fact grown over the prior decade. The Sandiford study was limited by being based purely on routinely collected health administrative data and the ARCOS studies are limited to the urban Auckland population—the largest metropolitan area in Aotearoa. However, M$\bar{\text{a}}$ori population density is greatest in non-urban areas.

The REGIONS care study used both prospectively collected patient level and health administrative data for the entire country to allow for inter-regional comparison and geographic adjustments. It also collected detailed patient level demographic, risk factor, pre-morbid function, stroke severity, and service provision data, which offered the ability to control for relevant potential confounders. The adjustment for stroke severity is a particularly important addition, as M$\bar{\text{a}}$ori presented not only at a younger age but also with more severe strokes on average which is an important predictor of outcome (Thompson S. G. et al., 2022). Stroke severity is difficult to assess in administrative data and thereby limits interpretation.

REGIONS care found differences between M$\bar{\text{a}}$ori and non-M$\bar{\text{a}}$ori for outcomes including functional independence (mRS 0–2) at 3 and 12 months [aOR 0.66 (0.42–1.00) and 0.59 (0.35–0.96), respectively] and death at 12 months [1.76 (1.07–1.89)]. The sub-study, using health administrative data, increased the study power and controlled for socioeconomic factors (Denison et al., 2023). Among 762 M$\bar{\text{a}}$ori and 6,117 non-M$\bar{\text{a}}$ori stroke patients M$\bar{\text{a}}$ori were more likely to have an increased odds of death at 3,6, and 12 months with ORs 1.5–1.7 (1.2–2.1) and these findings persisted after controlling for socioeconomic deprivation.

## Barriers to access

There is strong evidence reporting barriers for M$\bar{\text{a}}$ori to engage with health services. Some of these are due to fiscal constraints including travel, prescription, provider, and childcare costs as well as insecurity around taking time off work. However, socioeconomic factors are not the sole explanation for access inequities as differences persisted post-adjustment. Surveys and focus groups with M$\bar{\text{a}}$ori, who have lived experience of stroke, identified other barriers to quality stroke care and equitable outcomes such as ineffective communication, missed opportunities for family support, concern around being dismissed by health professionals resulting in delayed diagnosis, and reduced access to post-discharge support services in rural areas (Harwood et al., 2022; Thompson S. et al., 2022; Ranta et al., 2023). Some of these barriers have also been described in other parts of the health sector and are generally attributed to ongoing racism, stigmatization, culturally unsafe treatment environments, and culturally incongruent treatment approaches (Harris et al., 2006a,b).

## Proposed solutions

The stroke inequities described here are unfair, unjust, unacceptable and amenable to intervention. Many of the issues are not new and are also observed in other areas of health care (Ministry of Health & University of Otago, 2006). It is clear that more of “the same” will only maintain the status quo and therefore novel approaches are needed.

**Figure 1:**
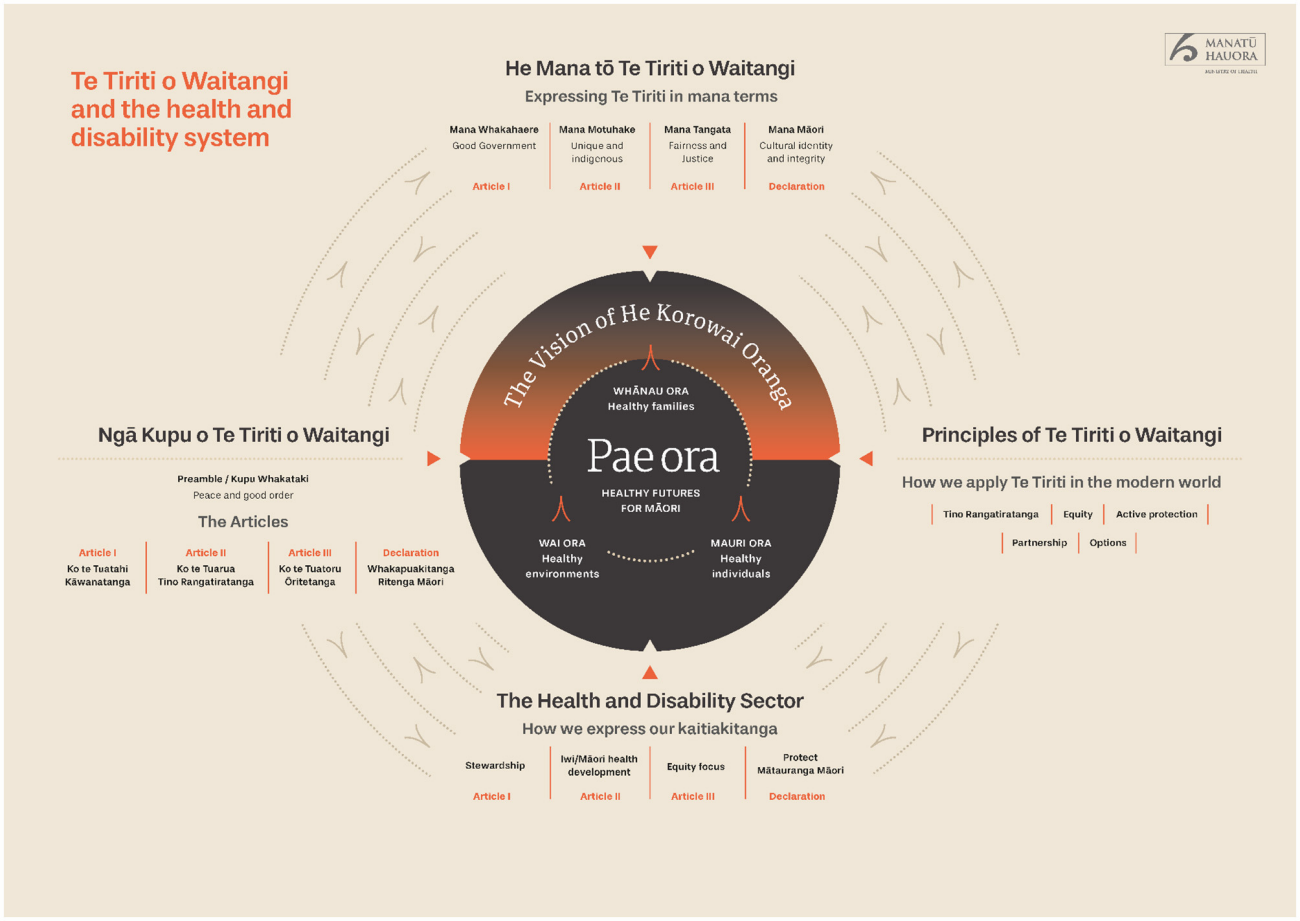
Te tiriti o waitangi and the aotearoa health system (Ministry of Health, 2020). Reproduced with permission. The diagram has not been altered from its original version, however, a second page with more detailed description has been truncated and can be found by following the link provided (Ministry of Health, 2020).

Premised on the Wai2575 Claim (Waitangi Tribunal Inquiry Claim Wai2575, 2023), the recent health system reform in Aotearoa aims to realize M$\bar{\text{a}}$ori rights to good health care and outcomes. Five key objectives are proposed—equity, tino rangatiratanga (self-determination), options, partnership and active protection of those rights. Taking a strengths-based approach, the emphasis is on prevention and general wellbeing rather than reactively starting “health care” after a person becomes unwell. This new approach also needs to incorporate traditional M$\bar{\text{a}}$ori knowledge and practices, including traditional healing (rongo$\bar{\text{a}}$) and a holistic approach that incorporates not only physical and mental aspects, but also wairua (spiritual) and wh$\bar{\text{a}}$nau (extended family) wellbeing (Figure 1).

Solutions to address current inequities include for M$\bar{\text{a}}$ori research to be primarily lead and executed by M$\bar{\text{a}}$ori themselves to ensure the most appropriate research questions are asked, the most appropriate intervention is designed and tested, and the right outcomes are assessed. Often partnering with non-M$\bar{\text{a}}$ori researchers is useful or even required, especially in areas where subject matter expertise, research capacity, and capability are still being developed. In these settings, non-M$\bar{\text{a}}$ori researchers and communities should ensure that service improvement and research activities are based on a genuine Te Tiriti o Waitangi partnership abiding by the principles outlined above. Using research guidelines such as the CONSIDER guideline, is also a very useful strategy to ensure M$\bar{\text{a}}$ori research, and indigenous research more broadly, is executed in a safe and effective fashion (Huria et al., 2019). Another key strategy is to consistently apply Kaupapa M$\bar{\text{a}}$ori research methodologies when investigating “M$\bar{\text{a}}$ori living with stroke.” Kaupapa M$\bar{\text{a}}$ori Research methodology ensures research employs principles that privilege the aspirations and priorities of M$\bar{\text{a}}$ori and respect the importance of including tikanga (traditional customs) in all aspects of the research (Smith, 1999). Kaupapa M$\bar{\text{a}}$ori Research is an indigenous, transformative methodology that is strengths-based and focuses on issues that need M$\bar{\text{a}}$ori-designed and M$\bar{\text{a}}$ori-led solutions. This approach supports the generation of knowledge by local M$\bar{\text{a}}$ori communities and translation into equitable outcomes for health and wellbeing of those M$\bar{\text{a}}$ori communities. To ensure research processes are culturally safe they should be guided by frameworks such as the Wh$\bar{\text{a}}$nau Tuatahi Research principles (Jones et al., 2010). This framework enables a practical adaptation of Westernized research methods within a Kaupapa M$\bar{\text{a}}$ori methodology and is informed by the following concepts: whakawhirinaki (trust), whakawhanaungatanga (building relationships), whakamana (empowerment), ng$\bar{\text{a}}$wari (flexibility), utu (reciprocity), and hurihuringa (reflexivity). Throughout all aspects of M$\bar{\text{a}}$ori directed research, M$\bar{\text{a}}$ori opinions and preferences need to be prioritized in setting directions, informing interventions, interpreting results and designing evaluations. This approach differs from the traditional “clinical expert” driven approaches which do not offer a culturally appropriate, proactive opportunity for M$\bar{\text{a}}$ori to contribute to co-designing solutions that will work for them.

An exemplar of how to operationalize this approach is the “Take Charge after Stroke” intervention. This intervention was developed using Kaupapa M$\bar{\text{a}}$ori methodologies including iterative co-design employing focus groups involving M$\bar{\text{a}}$ori with lived experience of stroke (Harwood, 2012). The resultant intervention involved patient-centered goal setting led primarily by non-clinicians allowing the patient to self-determine their treatment goals that could be focused on a number of motivational outcomes—not necessarily individual or physical in nature. Randomized controlled trial data proved outcomes were improved in M$\bar{\text{a}}$ori and Pacific peoples with stroke (Harwood et al., 2012a) and subsequent similar trial results showed the same benefits for people of NZ European ethnicity (Fu et al., 2020). This emphasizes that “what works for M$\bar{\text{a}}$ori also works for non-M$\bar{\text{a}}$ori” offering broad societal benefits. A key step is to promote widespread implementation of this and similar interventions that are underpinned by the appropriate research methodologies. Our team are currently using a similar co-design Kaupapa M$\bar{\text{a}}$ori methodology to develop additional stroke interventions in partnership with M$\bar{\text{a}}$ori health providers to specifically improve stroke outcomes for rural M$\bar{\text{a}}$ori.

Kaupapa M$\bar{\text{a}}$ori research also explores how M$\bar{\text{a}}$ori define “good outcomes” and whether Western models of physical independence (e.g., modified Rankin Scale) are aligned with M$\bar{\text{a}}$ori priorities and goals. Novel stroke outcomes have been developed to suit M$\bar{\text{a}}$ori and need to be more actively considered in M$\bar{\text{a}}$ori stroke research (Harwood et al., 2012b). Previously, quantitative Western research methodologies have often been imposed on M$\bar{\text{a}}$ori research projects where traditional Purakau (M$\bar{\text{a}}$ori storytelling) and other qualitative approaches may be better suited to gather the community insights and also develop interventions that will be acceptable and effective for various M$\bar{\text{a}}$ori communities. Scalability is sometimes challenging while preserving local ownership and M$\bar{\text{a}}$ori methodologies may seem inefficient and “lower quality” in comparison to Western hegemony. However, what has been tried to date has been ineffective and an indigenous approach placing tino rangatiratanga back in the control of M$\bar{\text{a}}$ori needs to be seriously considered—even if it requires a leap of faith for Western scientists. Trust is a two-way street and those whose trust has not been repeatedly betrayed ought not to hesitate to take the first step. The establishment of a new Aotearoa M$\bar{\text{a}}$ori Health Authority, Te Aka Whai Ora, is an important first step in shared decision-making for health services. Recent changes to grant assessment criteria, by national health research funding agencies such as the Health Research Council of New Zealand, now places more emphasis on M$\bar{\text{a}}$ori responsiveness and research methodologies, which is another significant positive development in signaling the importance of M$\bar{\text{a}}$ori health advancement.

A useful contribution for non-M$\bar{\text{a}}$ori New Zealanders is to engage in self-reflection exploring potential unconscious bias, acquiring cultural competencies to support a more culturally safe care environment for their M$\bar{\text{a}}$ori patients, and to learn about te ao M$\bar{\text{a}}$ori (the M$\bar{\text{a}}$ori world-view) to better appreciate the many benefits that a holistic, wellness-focused and strength-based approach brings to health outcomes for all. Placing greater importance on aspects such as spiritual wellbeing are not unique to M$\bar{\text{a}}$ori. In our depersonalized, institutionalized Western healthcare settings, we believe there is much to learn from indigenous practices that strongly support the notion of new approaches based in traditional practices that see individuals from a holistic perspective that values collective roles, responsibilities and outcomes, such as wh$\bar{\text{a}}$nau and community, above individual physical “health.”

As will undoubtedly be covered in other articles in this research topic, similar inequities have been reported for other Indigenous peoples across the globe. Indigenous Australians, North American, and other Pacific peoples, among others, have been reported to experience higher stroke incidence, stroke at a younger age, higher prevalence of stroke risk factors, and/or worse stroke outcomes including mortality (Atzema et al., 2015; You et al., 2015; Balabanski et al., 2018; Sanchez et al., 2021; Ogasawara et al., 2022). A comprehensive review of stroke incidence among Indigenous peoples in high income countries is currently underway (Balabanski et al., 2021). The issue of colonization including secondary socioeconomic disadvantage, discrimination, imposition of a “Western” world view and medical paradigm, and greater need for Indigenous self-determination have also been raised as ongoing challenges in populations outside of Aotearoa (The Lancet, 2020). While cultures are clearly different across the globe and locally informed and driven initiatives are certainly a key aspect in Aotearoa and likely also in other places, some synergies may well exist and it stands to reason that a much stronger collaborative network across Indigenous people with stroke and those providing stroke care, working on service improvement, and conducting relevant research with the aim of improving stroke outcomes for Indigenous People locally and potentially globally could enhance our collective efforts.

Recently members of the Aotearoa National Stroke Network comprising clinicians, health managers, and people with lived experience of stroke participates in a noho marae. This involved spending a night together at a M$\bar{\text{a}}$ori marae (traditional complex of buildings where discussion and formalities take place based on local, tribal practices) to strengthen connections and immerse themselves in a M$\bar{\text{a}}$ori environment resulted in a desire by one of the leaders with lived experience of stroke to increase connections with others around the globe to share experiences and learn from one another. A goal was set to work toward an Indigenous Stroke Conference in 2028 and anyone interested in contributing to this or any other potential areas for collaboration is encouraged to make contact with us.

## Conclusion

There are encouraging recent developments in Aotearoa that promise new opportunities using novel approaches developed “by M$\bar{\text{a}}$ori for M$\bar{\text{a}}$ori” to address current Indigenous stroke inequities. Greater global collaborations with indigenous stroke clinicians, researchers and indigenous people with lived experience of stroke are likely to be able to offer additional exciting opportunities and contribute to achieving equitable health outcomes for indigenous people.

## Author contributions

AR drafted the manuscript. MH and BJ provided critical review and revisions. All authors approved the final manuscript and take full responsibility for its content.
